# Identifying functional cortical plasticity after spinal tumour resection using navigated transcranial magnetic stimulation

**DOI:** 10.1308/rcsann.2024.0040

**Published:** 2024-07-04

**Authors:** L Onyiriuka, JM Aliaga-Arias, S Patel, A Khan, K Ashkan, R Gullan, R Bhangoo, A Ahmed, G Grahovac, F Vergani, A Kailaya-Vasan, JP Lavrador

**Affiliations:** ^1^ King’s College Hospital NHS Foundation Trust, UK; ^2^ University of Brescia, Italy; ^3^ King’s College London, UK

**Keywords:** Transcranial magnetic stimulation, Neuroplasticity, Intradural extramedullary tumour, Meningioma, Paraparesis

## Abstract

Our aim was to investigate the effectiveness of navigated transcranial magnetic stimulation (nTMS) brain mapping to characterise preoperative motor impairment caused by an intradural extramedullary (IDEM) tumour and postoperative cortical functional reorganisation. Preoperative and 1-year follow-up clinical, radiological and nTMS data from a case of thoracic spinal meningioma that underwent surgical resection of the lesion were collected and compared. A 67-year-old patient presented with severe progressive thoracic myelopathy (hypertonic paraparesis, clonus, insensate urinary retention) secondary to an IDEM tumour. Initial nTMS assessment showed bilateral upper limb representation with no positive responses for both lower limbs. He underwent successful surgical resection for his IDEM (meningioma WHO grade 1). At 1-year follow-up, the patient’s gait was improved and his bladder function normalised. nTMS documented positive responses for both upper and lower limbs and a decrease in the area (right side: 1.01 vs 0.39cm^2^; left side: 1.92 vs 0.81cm^2^) and volume (right side: 344.2 vs 42.4uVcm^2^; left side: 467.1 vs 119uVcm^2^) of cortical activation for both upper limbs, suggesting a functional reorganisation of the motor areas after tumour resection. nTMS motor mapping and derived metrics can characterise preoperative motor deficit and cortical plasticity during follow-up after IDEM resection.

## Background

Navigated transcranial magnetic stimulation (nTMS) is a non-invasive method of brain stimulation routinely employed in neurological and neurophysiological clinical practice; it has been gaining popularity in neurosurgery. nTMS has diagnostic and therapeutic applications. In surgical neuro-oncology, nTMS for motor mapping is employed in preoperative planning before tumour resections in close proximity to or infiltrating the primary motor cortex. Its ability to evaluate motor cortical excitability has prognostic value for postoperative outcomes and is associated with tumour grading of diffuse glioma.^1,2^ The activated cortical motor areas can be employed as seeding regions for corticospinal tractography and guide the surgical resection.

In patients affected by spinal cord injury (SCI), transcranial magnetic stimulation (TMS)-elicited motor-evoked potentials (MEP) represent an essential diagnostic tool, providing information about the functionality of intracortical neuronal structures and the electrophysiological integrity of the corticospinal tract, nerve root and motor pathways in the peripheral nervous system.^3–8^ In the therapeutic setting, TMS brain mapping has provided valuable insight into the physiological changes taking place at the cortical level that underly the therapeutic effects observed with TMS-based rehabilitation techniques.^9–16^

In a recent study, Zdunczyk *et al* demonstrated the potential role of nTMS in characterising functional disease stages in degenerative cervical myelopathy. They explored the concept of functional activation of a secondary motor area as an initial adaptive mechanism of continuous spinal cord compression and introduced the concept of corticospinal reserve capacity.^17^ In this report, we elaborate further on this concept to assess nTMS as a tool to measure functional cortical plasticity after resection of an intradural extramedullary (IDEM) spinal tumour.

## Methods

Clinical, radiological and nTMS data were collected prospectively from a clinical case of thoracic spinal meningioma causing progressive myelopathy and paraparesis that underwent surgical resection of the lesion. TMS assessments were performed to evaluate the degree of corticospinal tract damage and recovery in the preoperative setting and at 1-year follow-up, using the NEXSTIM® device. The patient provided informed consent for the procedures and use of clinical data for research purposes. No ethical approval was required because we applied the same protocol previously approved for preoperative mapping for tumour surgery.

The nTMS protocol consisted of: (1) rough mapping of the motor cortex to detect the cortical hotspots for upper and lower limb motor representation on the precentral gyrus on both hemispheres; (2) estimation of the resting motor threshold (RMT, the percentage of TMS machine power required to elicit an MEP of at least 50mA, at least 50% of the times that intensity is used) of each upper and lower limb hotspot; and (3) brain motor mapping of the four limbs at 105% of the determined RMT intensity. MEPs were detected, displayed on air and digitally recorded, as acquired from EMG recording of five muscles from each side of the body: abductor pollicis brevis, first dorsal interosseus, abductor digiti minimi, tibialis anterior and extensor hallucis longus.

Cortical activation maps were created using TMSMap Software®, estimating the area and volume of cortical representation from the data recorded in the nTMS protocol for the bilateral upper and lower limbs, at the preoperative stage and at 1-year follow-up.

To illustrate functional dissection of the cortical motor pathway, nTMS-seeded, enhanced constrained spherical deconvolution (CSD) tractography (Medtronic S8 Software®) was performed for the corticospinal tract (CST) from the preoperative and follow-up TMS data using positive nTMS sites as the cortical starting region of interest (ROI) and a mid-ROI placed in the brainstem and the level of decussation of the superior cerebellar peduncles.

A comparative assessment of the preoperative and follow-up clinical, radiological, nTMS, TMSMap and CSD tractography is reported.

## Case history

A 67-year-old male presented with progressive paraparesis over 4 years, until he was forced to mobilise using a wheelchair 3 weeks prior to presentation. The decline in mobility was associated with new mid-thoracic sensory changes, urinary symptoms (urgency, incomplete emptying, weak stream, incontinence) and saddle sensory loss.

On examination, he had a T4 sensory level with bilateral leg weakness (2/5 proximally on hip flexors, distally 4/5 in knee and ankles on Medical Research Council scale), hypertonia (grade 2 on the Modified Ashworth Scale), clonus (grade of 4+ National Institute of Neurologic Disorders and Stroke) and insensate urinary retention with a post-void bladder scan of 600ml, designated D on the American Spinal Injury Association Impairment Scale.

**Figure 1:**
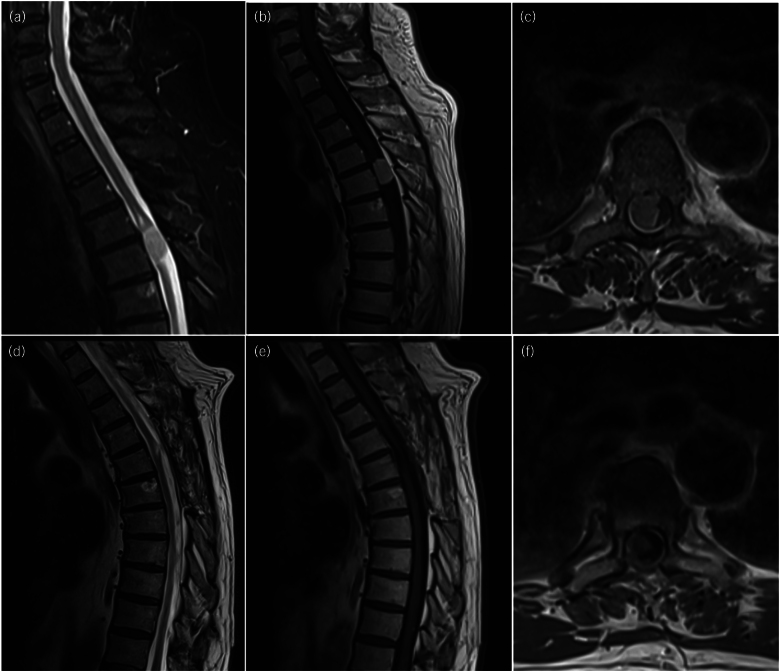
(a–c) Preoperative T2-weighted (a), T1 post-gadolinium (b) sagittal and T1 post-gadolinium axial (c) magnetic resonance imaging (MRI) scans documenting a mid-thoracic meningioma measuring 22×14×11mm in the cranio-caudal, anterior–posterior and latero-lateral axis, respectively. The tumour showed an intermediate T1 and T2 signal with avid homogenous enhancement and a small dural tail at the superoventral aspect. At lesion level there was effacement of the cerebrospinal fluid space and the spinal cord appeared displaced to the left, flattened and distorted, with signal abnormality extending between the levels of T4 and T5, suggestive of myelopathic changes. (d–f) T2-weighted (d) and T1 post-gadolinium (e) sagittal and axial T1 post-gadolinium (f) MRIs one year after surgery, documenting gross total resection of the lesion and absence of recurrence.

**Figure 2:**
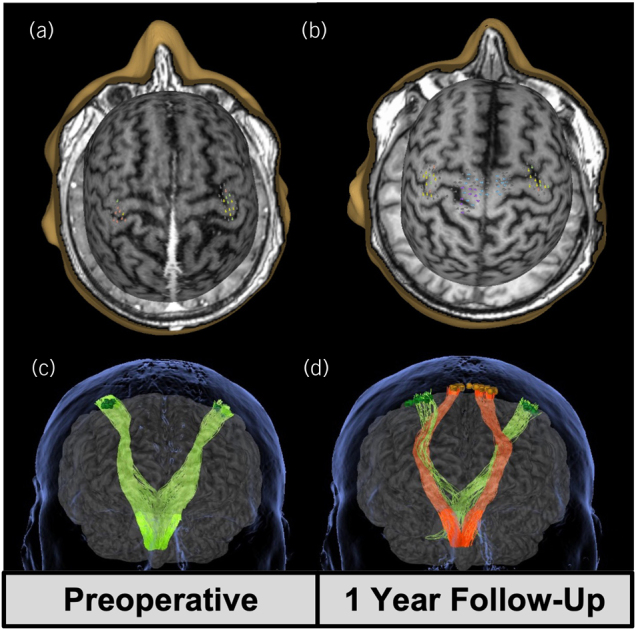
(a, b) Preoperative (a) and 1-year follow-up (b) navigated transcranial magnetic stimulation (nTMS) brain mapping showing recovery of the active motor representation of the lower limbs bilaterally. Grey dots = negative responses; yellow dots = abductor pollicis brevis muscle; orange dots = first dorsal interosseous muscle; green dots = abductor digiti minimi muscle; blue dots = anterior tibialis muscle; purple dots = extensor hallucis longus muscle. (c,d) Concordantly, preoperative (c) and 1-year follow-up (d) enhanced constrained spherical deconvolution tractography of the cortical spinal tract using positive nTMS sites as cortical starting region of interest (ROI) and a mid-ROI placed in the brainstem and the level of decussation of the superior cerebellar peduncles. Green tract = upper limb nTMS-seeded tractography; orange tract = lower limb nTMS-seeded tractography.

Spine magnetic resonance imaging (MRI) showed a well-defined IDEM lesion occupying a large proportion of the right side of the vertebral canal at the level of T4–T5 (Figure 1a–c). In preoperative motor mapping using nTMS, the RMTs were 33% for the left hand and 34% for the right hand. MEPs were not elicitable in the lower limbs (Figure 2a), therefore no RMTs could be estimated at that level.

The patient underwent surgical excision of the lesion through T4–T6 thoracic laminectomy, with intraoperative neurophysiological monitoring (IOM). D-wave recordings remained stable throughout the surgery and improvement in IOM parameters was apparent after spinal cord decompression. Histology revealed a meningioma, WHO grade 1, with very low Ki67 proliferation index (estimated 2%). Postoperative imaging confirmed a gross total resection of the lesion.

The preoperative neurological condition remained unchanged early after surgery. At day 5 after surgery, the patient was transferred to a neurorehabilitation unit. One year after surgery, gait was improved, with autonomous ambulation for short distances. Bladder and bowel dysfunction had resolved. The back pain was improved and radiculopathy resolved. The concomitant follow-up MRI showed no recurrence of the lesion (Figure 1d–f).

nTMS mapping performed at 1-year follow-up demonstrated elicitable MEPs for the lower limbs (Table 1) compared with no response preoperatively. The right foot had an RMT of 72% and 25 positive responses. The left foot had an RMT of 65% and 9 positive responses. RMTs of the hands were increased with respect to the preoperative test (38% on the right and 36% on the left) and fewer positive responses were elicited (12 on the right and 16 on the left) (Figure 2b).

**Table 1:** Navigated transcranial magnetic stimulation characteristics of the motor mapping and derived areas and volumes of activation, before surgery and at 1-year of follow-up

	RMT (%)	Positive responses	Area of activation (cm^2^)	Volume of activation (uVcm^2^)
Before surgery				
Right upper limb	34	30	1.01	344.2
Left upper limb	33	11	1.92	467.1
Right lower limb	–	–	–	–
Left lower limb	–	–	–	–
One year follow-up				
Right upper limb	38	12	0.39	42.4
Left upper limb	36	16	0.81	119
Right lower limb	72	25	0.15	11.2
Left lower limb	65	9	1.1	144.8

RMT = resting motor threshold

**Figure 3:**
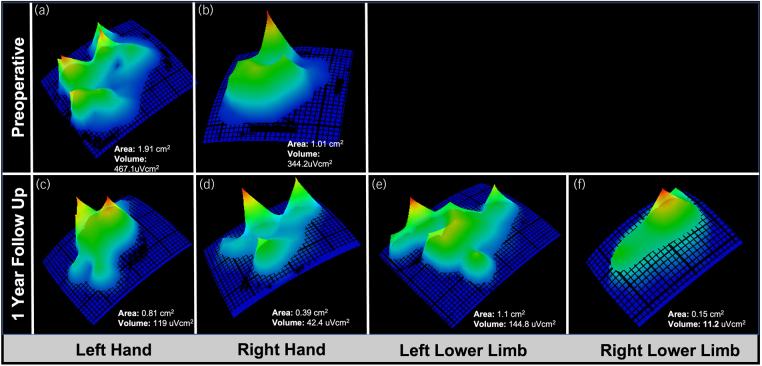
Area and volume of cortical activation bilaterally in the hand and lower limb preoperatively (a and b) and at 1-year follow-up (c–f). Functional activation of the hand is defined as joined activation of abductor pollicis brevis, first finger dorsal interosseous and abductor digiti minimi muscles*.* Functional activation of the lower limb is defined as joined activation of the anterior tibialis and extensor hallucis longus muscles*.* Images and measurements were made using TMSMap Software®.

Assessment of the preoperative and follow-up cortical activation maps showed recovery of the area and volume of cortical representation of the lower limbs, and of the feet, 1 year after surgery (Figure 3). The upper limbs showed a reduction in the estimated areas and volumes of activation (Table 1).

nTMS-seeded CSD tractography performed for the CST from the preoperative and 1-year follow-up TMS data, designed the corticospinal projection for the upper limbs at both stages. Lower limb functional subcortical anatomy that could not be identified before surgery was shown by the tractography seeded from the follow-up nTMS data (Figure 2c,d).

## Discussion

Given the impact on quality of life associated with spinal myelopathy caused by neuro-oncological disease, an integrated and comprehensive approach to the patient is always recommended. To the best of the authors' knowledge, this is the first report documenting the use of nTMS mapping for the assessment of preoperative and postoperative motor function in myelopathy secondary to a spinal tumour.

In concordance with previous literature findings in traumatic and degenerative myelopathy, the nTMS brain mapping data reported for this case coherently depicted the functional consequences on corticospinal excitability and motor cortical representation. At presentation, no MEPs were elicitable in the lower limbs and the clinical correlate was a preoperative paraparesis. We propose that a profound exhaustion of the cortical reserve capacity, as described by Zdunczyk *et al*, would account for the absence of elicitable MEPs by nTMS in this case of thoracic myelopathy caused by an extrinsic central nervous system tumour.^17^

Considering that recovery from SCI is known to take place as long as 1 year after the insult occurs, in the present case we performed the follow-up nTMS mapping 1 year after surgical removal of the spinal meningioma, to document the corticospinal excitability changes underlying the long-term clinical course. By that time, the patient had experienced a significant recovery of the strength in the lower limbs, with autonomous ambulation and reported symptomatic improvement of radicular and back pain. Concordantly, the nTMS was then able to elicit lower limbs MEPs.

Previous functional MRI, TMS and positron emission tomography studies have demonstrated extensive variations in cortical activation associated with movement impairment caused by central nervous system damage, showing expansion of cortical areas corresponding to muscles spared after SCI into cortical areas previously associated with muscles below the level of the lesion.^18^ The number of positive responses for the upper limbs decreased markedly 1 year after surgery compared with the preoperative results. Congruently, the estimated areas and volumes of cortical activation representing the upper limbs showed a radical decrease in size. These findings could depict the reversal of a cortical plasticity modulation initially induced by the cord damage provoked by the tumour, as reported previously in non-oncological conditions.^19–21^ The thoracic cord compression and associated oedema could suggest a hypothetical explanatory mechanism underlying the initial increase in cortical representation of the upper limb: when the electrical neural signal reaches the medullary alteration, a pathological antidromic neural transmission could recruit the motor units above the level of the lesion. Together with the synergistic effect that deafferentation from the structures below the level of the cord lesion is known to have on cortical excitability and plasticity, these events might have collectively produced an increased estimation of the cortical representation of the upper limbs in the nTMS mapping.

Similarly, different functional cortical activations may translate in different subcortical functional dissections of the CST using nTMS-seeded tractography (enhanced spherical deconvolution in the current case). The postoperative functional dissection of the upper limb was decreased with respect to the tract depicted from the preoperative nTMS data, in accordance with the changes of nTMS values reported above. A partial volumetric over-imposition of the preoperative estimation of the CST for the upper limb with the follow-up estimated projection from the lower limb could be referred to an artefactual tract processing of the spherical deconvolution technique employed. Although a functional significance of this finding remains doubtful, there is a possibility that such CST representation is related to an incomplete preoperative axonal retrograde degeneration of the tract related with the lower limb. Also, we hypothesise that a potential increase in recruitment of CST fibres for upper limb motor control, either from other areas of the primary motor cortex (in the absence of activation related with the lower limb) or non-motor neurons, can occur.^17^

## Conclusion

This case report supports the effectiveness of nTMS in the evaluation of functional cortical plasticity in spinal conditions. Successful motor evolution after IDEM tumour resection is associated with recovery of nTMS responses and normalisation of functional cortical representation of motor areas.
